# Depression and multiple sclerosis: A bidirectional Mendelian randomisation study

**DOI:** 10.1177/1352458521996601

**Published:** 2021-02-19

**Authors:** Stefanie Binzer, Xia Jiang, Jan Hillert, Ali Manouchehrinia

**Affiliations:** Department of Clinical Neuroscience, Karolinska Institutet, Stockholm, Sweden/Department of Neurology, Kolding Hospital, Kolding, Denmark; The Neuroepidemiology Research Group, Stockholm, Sweden; Department of Clinical Neuroscience, Centre for Molecular Medicine, Karolinska Institutet, Stockholm, Sweden; Department of Clinical Neuroscience, Karolinska Institutet, Stockholm, Sweden/Karolinska University Hospital, Stockholm, Sweden; Department of Clinical Neuroscience, Karolinska Institutet, Stockholm, Sweden/The Karolinska Neuroimmunology and Multiple Sclerosis Centre, Centre for Molecular Medicine, Karolinska Institutet, Stockholm, Sweden

**Keywords:** Genetics, multiple sclerosis

## Abstract

Depression is common in multiple sclerosis (MS); however, the underlying mechanism for the relationship remains unknown. In this study, we examined a putative causal relationship between depression and MS using a bidirectional Mendelian randomisation (MR) framework. Using the latest genome-wide association study data available, 168 non–major histocompatibility complex (MHC) independent variants associated with MS and 96 independent genetic variants associated with depression susceptibility were used. Maximum likelihood, weighted median, inverse variance weighted method and MR-Egger regression analyses were performed. There was no significant risk for the development of MS in persons carrying variants associated with depression or for risk of depression in individuals who are genetically susceptible to MS.

## Introduction

It has been shown that the risk of depression is substantially increased in persons with multiple sclerosis (MS) with increased Incidence of up to 71% compared to control population.^1^ Recent studies have also found that concurrent depression is associated with risk of disability worsening in those affected by MS.^2^ The underlying mechanism for the relationship between depression and MS still remains unknown. Central nervous system inflammation is shown to be part of both conditions which may indicate genetic similarity. However, Johansson et al.^3^ investigated depression in siblings of MS patients and concluded that genetic liability could not solely explain the association between MS and depression.

Mendelian randomisation (MR) is an approach that uses genetic variants as instrumental variables (IVs) for investigating the association between a risk factor and an outcome. It is designed based on the fact that genetic variants are randomly allocated during gamete formation and conception therefore are independent of confounding factors, which, makes the results less susceptible to reverse causality and confounding bias – difficulties often encountered in conventional observational studies.^4^ In MS, MR studies have been employed to investigate the causal associations between environmental and lifestyle factors such as body mass index (BMI) and vitamin-D levels and risk of MS.^5^ In this study, we performed a bidirectional MR analysis with the aim of investigating the causal association between depression and MS. We took advantage of the largest publicly available genome-wide association study (GWAS) data available for both MS^6^ and major depression disorder (MDD).^7^

## Materials and methods

### Data

#### GWAS data for MS

The most recent publication of the International Multiple Sclerosis Genetics Consortium which included more than 47,000 MS cases and 68,000 controls was used.^6^ In this GWAS, 233 statistically independent genome-wide significant single-nucleotide polymorphisms (SNPs) were found to be associated with MS susceptibility, explaining about 39% of the genetic predisposition to MS.^6^ Of the 231 variants, 200 are located outside of the major histocompatibility complex (MHC) region. The variants outside MHC region account for approximately 19% of the MS heritability.^6^ In this analysis, we only used the non-MHC variants for construction of MS IV due to MHC variants having significant pleiotropic effect which resulted in inclusion of 168 non-MHC independent variants.

#### GWAS data for depression

We used data from the most recent depression GWAS, where the authors meta-analysed data on 246,363 cases and 561,190 controls from the three largest GWAS of MDD with a phenotypic variance ranging from self-reported depression to depression identified in hospital records.^7^ A total of 102 independent SNPs associated with MDD were identified.^7^ Twin studies have provided heritability estimates of depression to be approximately 30%–40%,^8^ and it is estimated that proportion of phenotypic variance jointly accounted for by all additive genetic effects is approximately 9%.^9^ From the 102 SNPs associated with depression, a total of 96 matched the IVs used for MS.

### MR analysis

We used the *MendelianRandomization* package for MR analyses using *R* software (version 3.4; R Project for Statistical Computing, Vienna, Austria). We employed several MR approaches including inverse variance weighted (IVW), maximum likelihood and weighted median methods. We used MR-Egger to test for the presence of pleiotropy. The strength of IVs (F-statistic) which is the proportion of variance in the phenotype explained by sample size, number of IVs and genetic variance was also calculated. We furthermore searched the GWAS catalogue (https://www.ebi.ac.uk/gwas) to identify if any of the genetic variants had any established associations with other traits. We found that 17 SNPs which were previously reported to be associated with other diseases and/or traits including intelligence, cognitive ability, neuroticism, smoking, primary biliary cholangitis, BMI, obesity, thyroid peroxidase antibody positivity and risk-taking tendency. We thus performed sensitivity analysis excluding those pleiotropic SNPs. Furthermore, we performed a multivariable MR approach to adjust for potential horizontal pleiotropy, which can be acting through BMI, alcohol consumption, vitamin-D and smoking behaviour (four major lifestyle factors that are likely to confound the MDD-MS relationship). As palindromic SNPs can introduce ambiguity, all analyses were performed again after removal of palindromic SNPs. Given the current sample size, our study had 80% power to detect a modest effect of 6% (odds ratio = 1.06 or 0.94). However, if the true effect is even smaller than this – a magnitude that is probably of limited clinical relevance – our study was underpowered to detect.

No ethical approval was necessary for the publicly available data which is de-identified.

## Results

We did not find any significant association between depression and MS in any direction. The risk of MS in genetically susceptible individuals to depression was (odds ratio (OR): 1.03, 95% confidence intervals increased by 3% (CI): 0.80–1.34, *p* = 0.81) using the IVW method. The risk of depression in genetically susceptible individuals to MS was OR: 1.00 (95% CI: 0.99–1.01, *p* = 0.24; Table 1). Removal of palindromic SNPs and adjustment for BMI, smoking, vitamin-D and alcohol consumption did not change our results (Table 1). Exclusion of pleiotropic SNPs or SNPs associated with confounding traits as indicated by the GWAS catalogue also did not alter the findings.

**Table 1.** Risk of MS or depression in genetically susceptible individuals.

	#SNPs	OR (95% CI)	*p* value	*p* value for intercept	#SNPs	OR (95% CI)	*p* value	*p* value for intercept
	Full-set				Remove palindromic SNPs			
Risk of MS in persons genetically susceptible for depression								
IVW	96	1.03 (0.80–1.34)	0.81		83	1.03 (0.78–1.37)	0.83	
Maximum likelihood	96	1.03 (0.79–1.35)	0.81		83	1.03 (0.78–1.37)	0.82	
MR-Egger	96	0.79 (0.27–2.36)	0.68	0.63	83	0.62 (0.20–1.93)	0.41	0.36
Weighted median	96	1.06 (0.80–1.40)	0.69		83	0.99 (0.74–1.33)	0.94	
Adjusted for BMI	39	1.26 (0.77–2.05)	0.34					
Adjusted for smoking	79	1.02 (0.76–1.37)	0.38					
Adjusted for alcohol consumption	79	1.00 (0.75–1.34)	0.77					
Adjusted for vitamin-D	95	1.00 (0.76–1.31)	0.98					
Risk of depression in persons genetically susceptible for MS								
IVW	168	1.00 (0.99–1.01)	0.24		148	1.00 (0.99–1.01)	0.34	
Maximum likelihood	168	1.00 (0.99–1.01)	0.14		148	1.00 (0.99–1.01)	0.22	
MR-Egger	168	1.01 (0.98–1.04)	0.34	<0.001	148	1.01 (0.98–1.04)	0.37	<0.001
Weighted median	168	1.00 (0.99–1.01)	0.33		148	1.00 (0.99–1.01)	0.46	

IVW: inverse variance weighted; OR: odd ration; CI: confidence interval; BMI: body mass index

## Discussion

In this study, we used MR approach to examine the relationship between depression and MS. We took advantage of summary statistics from the most recent and also the largest GWAS conducted for both depression and MS. Hundreds of independent SNPs with genome-wide significance for depression and MS were used as IVs – these IVs constitutes strong instruments with *F*-statistics >700. We did not find evidence to support a causal genetic risk between depression and MS in either directions. We examined the possibility of pleiotropic effects via MR-Egger regression but did not find evidence of this. We searched the GWAS catalogue for SNPs associated with other traits, and exclusion of those did not have an effect on the results.

Our results are in line with previous studies finding that the association between MS and depression could not be explained by genetic liability alone.^3^ This provides evidence that other factors seem to account for the increased incidence observed between these two diseases.

Disorders of the brain can present considerable epidemiological comorbidity and often share clinical symptoms, provoking debates about their etiological overlap. A recent study conducted by the Brainstorm Consortium quantified the shared genetic basis of 25 brain disorders from GWAS of 265,218 patients and 784,643 control participants and found that while psychiatric disorders share common variant risk, neurological disorders appear more distinct from one another and from the psychiatric disorders.^10^ These findings were corroborated by our non-causal relationship identified between depression and MS, indicating distinct underlying pathogenic processes, at least on the genetic level.

Limitations of our study include the fact that the GWAS data are based on genetic material from mainly people with European ancestry (even though consistency of European ancestry for the exposure and outcome populations is actually a strength). Thus, our results might not be applicable to other population groups. Furthermore, the GWAS data used for depression encompass a wide range of phenotypes, thus results might change if a more stringent criteria for the diagnosis of depression are used.
